# Tri-Channel Electrochemical Immunobiosensor for Combined Detections of Multiple Exosome Biomarkers of Lung Cancer

**DOI:** 10.3390/bios12070435

**Published:** 2022-06-21

**Authors:** Cui Fan, Bingyan Jiang, Wenjia Shi, Dan Chen, Mingyong Zhou

**Affiliations:** 1 State Key Laboratory of High Performance Complex Manufacturing, Central South University, Changsha 410083, China; 193712133@csu.edu.cn (C.F.); jby@csu.edu.cn (B.J.); 2 College of Mechanical and Electrical Engineering, Central South University, Changsha 410083, China; 3 Department of Human Anatomy and Neurobiology, School of Basic Medical Science, Central South University, Changsha 410083, China; 206511017@csu.edu.cn (W.S.); chendan0101@csu.edu.cn (D.C.)

**Keywords:** electrochemical immunobiosensor, exosome detection, multiple biomarkers, lung cancer

## Abstract

Current methods for the early diagnosis of cancer can be invasive and costly. In recent years, exosomes have been recognized as potential biomarkers for cancer diagnostics. The common methods for quantitative detection of exosomes, such as nanoparticle tracking analysis (NTA) and flow cytometry, rely on large-scale instruments and complex operation, with results not specific for cancer. Herein, we present a tri-channel electrochemical immunobiosensor for enzyme-free and label-free detecting carcino-embryonic antigen (CEA), neuron-specific enolase (NSE), and cytokeratin 19 fragments (Cyfra21-1) from exosomes for specific early diagnosis of lung cancer. The electrochemical immunobiosensor showed good selectivity and stability. Under optimum experimental conditions, the linear ranges were from 10^−3^ to 10 ng/mL for CEA, 10^−4^ to 10^2^ ng/mL for NSE, and 10^−3^ to 10^2^ ng/mL for Cyfra21-1, and a detection limit down to 10^−4^ ng/mL was achieved. Furthermore, we performed exosome analysis in three kinds of lung cancer. The results showed a distinct expression level of exosomal markers in different types. These works provide insight into a promising alternative for the quantification of exosomal markers in specific diseases in the following clinical bioassays.

## 1. Introduction

Cancer has been the focus of scientific research over the past decades because of its high incidence rate and high mortality rate. Early diagnosis and effective treatment are two of the biggest challenges in the fight against cancer [1]. Statistically, lung cancer is the leading cause of cancer-induced mortality, with the highest incidence among cancers, and the highest mortality in males and the second in females [2]. With great achievements in surgery, radiotherapy, and chemotherapy, the survival rate of cancer patients has improved, but the five-year survival rate of lung cancer is still below 20%. Early diagnosis of lung cancer has become extremely important [3].

Exosomes are small (30–150 nm) membranous vesicles containing proteins, phospholipid bilayers, genetic material, and metabolites with abundant information from parental cells [4]. Cancer cell-derived exosomes carry information such as DNA, RNA, and proteins of the parent cancer cells. The basic information can be obtained directly by analyzing exosomes, because the expression profiles of exosomal nucleic acids and proteins are altered in many diseases, including cancer, demonstrating their promise as a noninvasive biomarker for early detection and diagnosis [5]. Yokoyama et al. [6] compared the detection results of serum tumor marker CEA with exosome surface carcinoembryonic antigen (exo-CEA) in 48 patients with colorectal cancer. As a tumor marker, the surface protein on the exosome has higher accuracy for clinical diagnosis. To date, most methods are based on exosome-specific marker antigens or proteins, such as CD63 [7,8,9,10,11], CD9 [12,13,14,15,16], EpCAM [17,18,19,20,21], etc. However, it is not specific to realize the early diagnosis of cancer by quantifying the classical markers of exosomes, because exosomes can be secreted by healthy cells and any tumor cells [22]. In this work, three markers closely related to lung cancer were selected, namely carcinoembryonic antigen (CEA), neuron-specific enolase (NSE), and cytokeratin 19 fragment (Cyfra21-1), which can significantly improve the detection specificity [23], and even have guiding significance for pathological typing [24]. As a new potential biomarker for tumor cells, exosome detection shows great promise for the diagnosis of cancer. However, reliable and effective approaches are still in demand to achieve the high sensitivity detection of exosomes [25].

An electrochemical biosensor utilizing electrochemical immunodetection methods has been widely reported in the literature. It has various advantages in the detection of exosomes due to the high sensitivity, good specificity, rapid response, and ease of integration [26,27,28]. Su et al. [29] developed a sensitive and portable electrochemical biosensor in combination with smartphones for quantitative analysis of exosomes. The biosensor could detect as low as 7.23 ng of CD63-positive exosomes in 5 μL of serum within 2 h, which promoted the application in point-of-care testing. The traditional immunoassay methods are labeled immunoassay and depend on electroactive materials and enzymes such as Hexaammineruthenium (III) chloride (Ru(NH_3_)_6_^3+^) [30], Prussian blue [31], horseradish peroxidase(HRP) [32], etc. However, these reported methods tent to have certain limitations, such as the increase in operation steps making it difficult to obtain stable signals. Therefore, label-free electrochemical immunoassays are considered to be one of the most promising methods on account of their easy operation and their ability to reduce the interference caused by the increase in operation steps [33]. Generally, electrochemical systems employ a three-electrode setup, including reference, counter, and working electrodes, in which the working electrode is the key part, highly interrelated with the detection results. The electrode materials used in research include glassy carbon electrodes (GCEs) [34], precious metal electrodes [35], screen-printed electrodes (SPEs) [36], and indium tin oxide electrodes (ITO) [37]. Compared with the traditional three-electrode system, the integrated system on a single chip has the advantages of small electrode size, easy storage, and convenience in use. The ITO glass electrode has the advantages of easy integration, low cost, and mass production compared to the gold electrode and the screen-printed electrode. Furthermore, micro-chambers in series can be integrated on the three-electrode to realize multiple detections instead of the beaker or manual dropping of samples, which could reduce the sample cost, control the flow rate, and decrease the background interference effects.

**Scheme 1:**
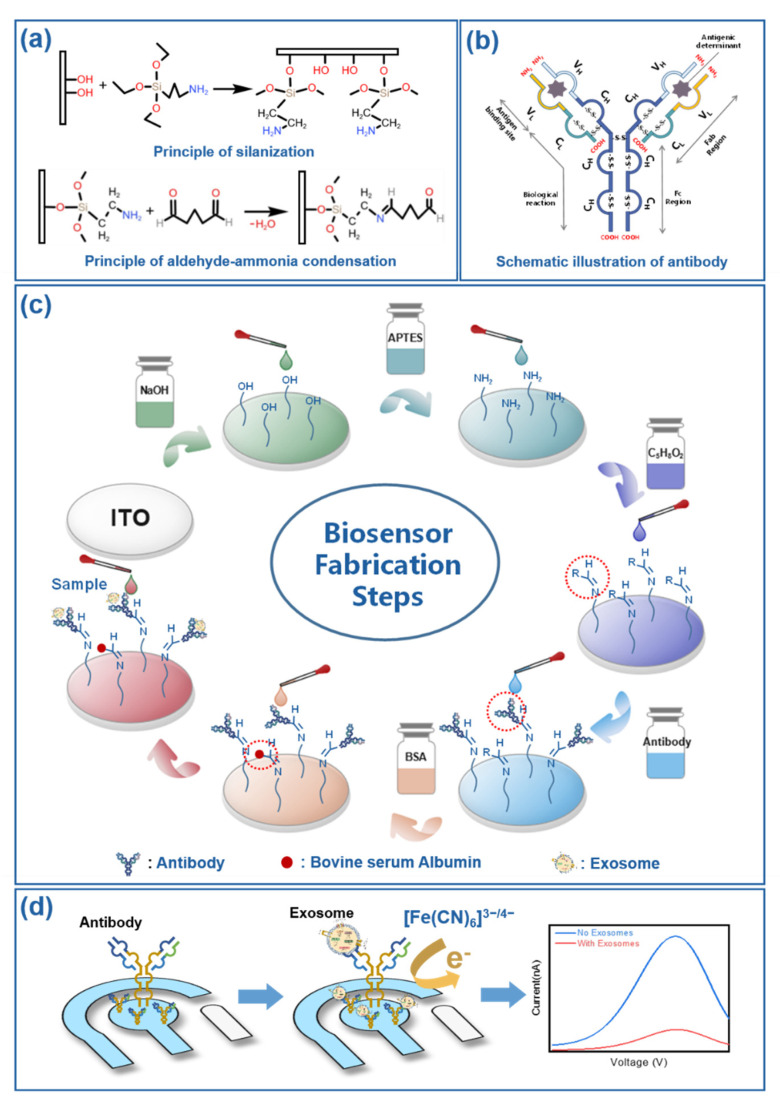
Schematic representation of the designed immunosensor for exosome biomarkers detection. (**a**) Principle of silanization process by APTES and aldehyde-ammonia condensation by glutaraldehyde on the hydroxyled working electrode, (**b**) Schematic illustration of antibody, (**c**) Fabrication procedures of the electrochemical immunosensor, (**d**) Principle of differential pulse voltammetry for the detection of exosomes.

Herein, leveraging the advantages of a three-electrode system that was combined with immunoassay method, we reported a tri-channel electrochemical immunosensor on ITO glass for enzyme-free and label-free detection of the multiple exosome biomarkers CEA, NSE, and Cyfra21-1, which are closely related to lung cancer. Scheme 1 shows the fundamental basis of the immunosensor in the detection of exosome biomarker. Once the cancer-specific exosomes were captured by the immunobiosensor, the immune complex formed by the combination of exosomes and antibodies hinders electron mass transfer; differential pulse voltammetry (DPV) was used to monitor the weak faradaic currents caused by the change of concentration, as shown in Scheme 1d. Under optimum experimental conditions, the multiple detections of exosome markers of three lung cancer cells were realized. The tri-channel electrochemical immunosensor shows great potential in the application for lung cancer early screening in the future.

## 2. Experimental

### 2.1. Materials and Reagents

Anti-CEA, Cy3 labeled anti-NSE, from Biosynthesis Biotechnology Co., Ltd. (Beijing, China) and anti-Cyfra21-1 from Anyan trade Co., Ltd. (Shanghai, China) were used in the experiments. Conductive indium tin oxide (ITO) glass substrate was purchased from Shenghua Technology Co., Ltd. (Guangzhou, China). Potassium ferricyanide (K_3_Fe(CN)_6_), (3-Aminopropyl) triethoxysilane (APTES), and Glutaraldehyde were obtained from Shanghai Aladdin Bio-Chem Technology Co., Ltd. (Shanghai, China). Potassium ferrocyanide (K_4_Fe(CN)_6_) and potassium chloride (KCl) were purchased from Sinopharm Chemical Reagent Co., Ltd. (Shanghai, China). Bovine Serum Albumin (BSA), fetal bovine serum (FBS), RPMI-1640 medium, and Dulbecco’s modified eagle medium (DMEM) were supplied by Biological Industries Co., Ltd. (Kibbutz Beit Haemek, Israel). Lipophilic green, fluorescent dye (Dio) was configured according to the manufacturer’s instructions (Biyuntian, China). All reagents used in the experiment were of analytical grade, and all solutions were prepared with Milli-Q ultrapure water (MilliQ, Millipore, MA, USA).

### 2.2. Fabrication of Tri-Channel Electrodes

**Figure 1:**
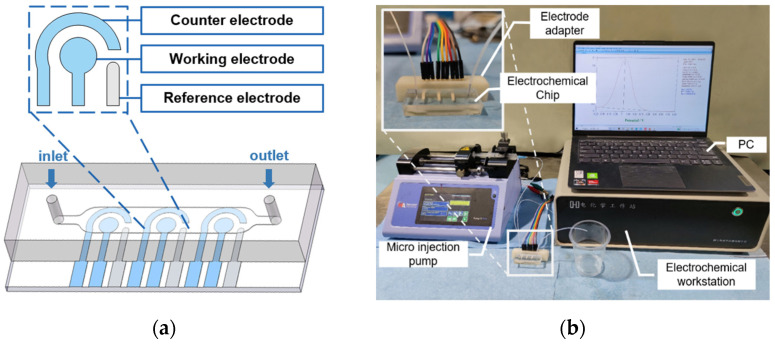
Schematic diagram of (**a**) the structure of electrochemical chip, (**b**) experimental setup.

Electrochemical detection techniques typically employ a classical three-electrode configuration consisting of a working electrode, reference electrode, and counter electrode, as shown in Figure 1a. An Indium tin oxides (ITO) thin film, a conductive transparent layer that was used as a working electrode, was deposited on the glass substrates. Three identical three-electrode sensor arrays were designed by AutoCAD and etched precisely by laser on a 1.1-mm-thick ITO glass substrate. The shape of the reference electrode was marked on the tape, and then the tape was pasted onto the surface of ITO glass. Ag/AgCl paste was brushed onto the whole of the tape and placed on a hot plate at 100 °C for 60 min before the tape is removed. A polydimethylsiloxane (PDMS) chip with micro-channel was prepared by the soft photolithographic process. After that, the chip was plasma-bonded to the surface of the glass side. Thus, a fully functional basic tri-channel three-electrode electrochemical system was prepared. Cyclic voltammetry scanning from −0.2 V to 0.6 V at 50 mV/s scan rate was applied to characterize the performance of the Ag/AgCl pseudo-reference electrode in 5 mM [Fe(CN)_6_]^3−/4−^ solution containing 0.1 M KCl.

### 2.3. Functionalization of Immunosensor

Antibodies are essentially proteins composed of amino acids, with amino and carboxyl groups at both ends of the antibodies, as seen in Scheme 1b. The surfaces of the ITO working electrodes were immobilized with antibodies by chemical crosslinking technology. The (3-Aminopropyl) triethoxysilane (APTES) acts as an intermediate medium that couples the hydroxyl group on the surface of the ITO to form a covalent bond, while exposing the amino group at the other end. The amino groups of antibody and APTES were condensed with the aldehyde group of glutaraldehydes, and antibody was immobilized by crosslinking with glutaraldehyde onto the working electrode. The chemical reaction equations are shown in Scheme 1a.

The fabrication procedure of the electrochemical immunosensor is schematically displayed in Scheme 1c. Firstly, the ITO glass was sequentially washed in the ultrasonic cleaner with isopropanol, deionized (DI) water, anhydrous ethanol for 10 min, and fresh mixed solution (NaOH: anhydrous ethanol = 1:1) for 5 min. After drying under nitrogen, the ITO glass was ammoniated with 1% APTES in anhydrous ethanol at 37 °C for 12 h and washed in the ultrasonic cleaner with anhydrous ethanol five times; it was then placed in an oven at 120 °C for 3 h. Next, the ITO glass was placed in 5% glutaraldehyde in PBS solution for 2 h at room temperature for effective immobilization of antibodies. After rinsing with phosphate buffer saline (PBS) and drying with nitrogen, anti-CEA, anti-NSE and anti-Cyfra21-1 solutions were respectively dripped onto the surfaces of three working electrodes and incubated at 37 °C for 1 h. Then, the electrode was washed three times with 10 mM PBS to remove any excess unbound antibodies. Finally, the electrode was incubated with 1% BSA for 1h to block unoccupied sites and was then washed with PBS. Water contact angle measurement (JC2000D, POWEREACH, Shanghai, China) was used to characterize the surface hydrophilicity after each step. At room temperature, a 3 μL deionized water drop was dropped onto the working electrode using a micro pipette. The water contact angle should be measured immediately after the droplet stabilizes, with each sample measured at least three times. Furthermore, electrochemical impedance spectroscopy (EIS) was applied to further indicate the binding of the ITO/Antibody/BSA/exosome onto the working electrode. A selective immunosensor was prepared and stored at 4 °C prior to the next step of capturing the exosomes to be measured and for the subsequent electrochemical detection in the presence of the [Fe(CN)_6_]^3−/4−^-KCl redox system.

### 2.4. Cell Culture and Isolation of Exosomes

#### 2.4.1. Cell Culture

Three lung cancer cell lines (NCI-H1395, NCI-H226, and NCI-H446, derived from lung adenocarcinoma, lung squamous carcinoma, and small cell lung cancer, respectively) were kindly provided by Cell Bank, the Chinese Acadamy of Science [38]. All cells passed tests for mycoplasma contamination and were cultured in RPMI 1640 supplemented with 10–20% exosome depleted fetal bovine serum (VivaCell, Shanghai, China) in a CO_2_ incubator (BPN-50CH, Yiheng Scientific Instrument, Shanghai, China) at 37 °C with 5% CO_2_. Cell recovery, cell passage, and cell cryopreservation processes were performed on a vertical superclean bench (SW-CJ-2D, Suzhou Purification Equipment, Suzhou, China), except for centrifugation (TGL-16M, Xiangyi Laboratory Instrument Development, Changsha, China) and the water-bath heating process (HH-2, Lichen Instrument Technology, Shaoxing, China).

#### 2.4.2. Extraction of Exosome

Cells were grown in T75 cm^2^ flasks to approximately 80% confluence, and then the medium was replaced with exosome-free FBS-supplemented RPMI 1640. After 24 h of starvation culture, cell culture supernatant was collected and centrifuged at 300× *g* for 10 min, 2000× *g* for 20 min, to discard cells and cellular debris, and 10,000× *g* for 30 min, to remove extracellular vesicles. The supernatant was transferred to a 100-kD ultrafiltration tube (Millipore, MA, USA), and centrifuged at 4000× *g* for 30 min, followed by extraction with Total Exosome Isolation Reagent (Invitrogen, Thermo Fisher Scientific, Waltham, MA, USA) from the above cell suspension. The exosome extraction procedure was carried out strictly according to the manufacturer’s protocol.

#### 2.4.3. Characterization of Exosomes

Transmission electron microscopy (TEM), nanoparticle tracking analysis (NTA), and fluorescence microscope were applied to characterize the morphology, concentration, and binding to antibodies of the exosomes, respectively. 

The preparation processes of the exosome sample for TEM using the negative staining technique are as follows. Freshly prepared 4% paraformaldehyde was mixed with an equal volume of exosome sample and was then pipetted dropwise into a 200-mesh copper grid for 20 min. Subsequently, two PBS washes were performed along with blotting with filter paper, followed by 5 min incubation with 1% glutaraldehyde in PBS to fix exosomes, washing five times with dH_2_O, and drying. The grid was then negatively stained with 2% phosphotungstic acid twice, protected from light for 3 min to enhance contrast. The morphology of the exosome was observed via TEM (Talos F200X, Thermo Fisher Scientific, Waltham, MA, USA) at an accelerating voltage of 80 kV after drying at room temperature.

NTA was employed to determine the size distribution and concentration of exosome extracted from the Invitrogen. Briefly, the exosome was diluted 50-fold to a concentration suitable for detecting with ZetaView PMX 110 (Particle Metrix, Meerbusch, Germany). The size distribution and concentration of samples were performed automatically, and the data were exported for further analysis.

Immunobiosensor was prepared by immobilizing Cy3-anti-NSE on the working electrode. Lipophilic green, fluorescent dye (Dio) was configured according to the manufacturer’s instructions. Exosomes were stained with the prepared Dio membrane dye in a ratio of 2:1 for 5–20 min and incubated with cy3-anti-NSE for 30 min; all steps were protected from light at 37 °C. The immobilization of the cy3-anti-NSE to the surface of the ITO and the immune binding of exosomes and cy3-anti-NSE were visualized using a fluorescence microscope (ECLIPSE Ti2-U, Nikon, Yuriage, Japan).

### 2.5. Electrochemical Detection

The sample to be tested was diluted in Tris-HCl buffer (10 mM, pH = 7.4). After incubation with antibodies at 37 °C for 30 min, the unbound sample was thoroughly washed away with the same buffer. Differential pulse voltammetry (DPV) is a label-free technique for investigating electrochemical signal changes in quick bioanalysis. It can reduce the interference caused by the charging current in the background current, and significantly improves the sensitivity [39]. The immune binding of antibodies and exosomes on the electrode will obstruct the electron transfer of the [Fe(CN)_6_]^3−/4−^, which causes a decrease in the peak current of the electrochemical response curve, as shown in Scheme 1d. Therefore, all electrochemical signals were measured by differential pulse voltammetry (DPV, voltage range: −0.3 to 0.2 V, pulse amplitude: 50 mV, pulse width: 50 ms) in the presence of the [Fe(CN)_6_]^3−/4−^-KCl redox system by using an electrochemical workstation (CHI600E, Chenhua, Shanghai, China). Figure 1 shows the schematic diagram of the structure of the electrochemical chip and the experimental setup.

## 3. Results and Discussion

### 3.1. Electrochemical Chip

**Figure 2:**
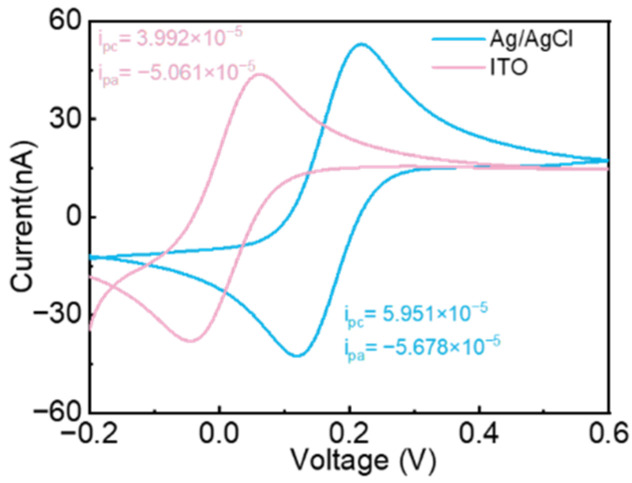
Cyclic voltammetry responses of 5 mM [Fe(CN)_6_]^3−/4^ solution containing 0.1 M KCl at bare ITO (pink) and Ag/AgCl reference electrode (blue).

The standard electrochemical detection system consists of a working electrode, a counter electrode, and a reference electrode. The material of the reference electrode has an impact on the stability of the detection system. In this work, Ag/AgCl was selected as the reference electrode. Cyclic voltammetry (CV) measurement was performed to compare the electrochemical reaction of the bare ITO electrode with the Ag/AgCl reference electrode. As shown in Figure 2, the ratio of the absolute values of the cathodic peak current density versus the anodic peak current density (|i_pc_/i_pa_|) were 0.789 and 1.048, respectively. The Ag/AgCl reference electrode demonstrated better redox reversibility. 

**Figure 3:**
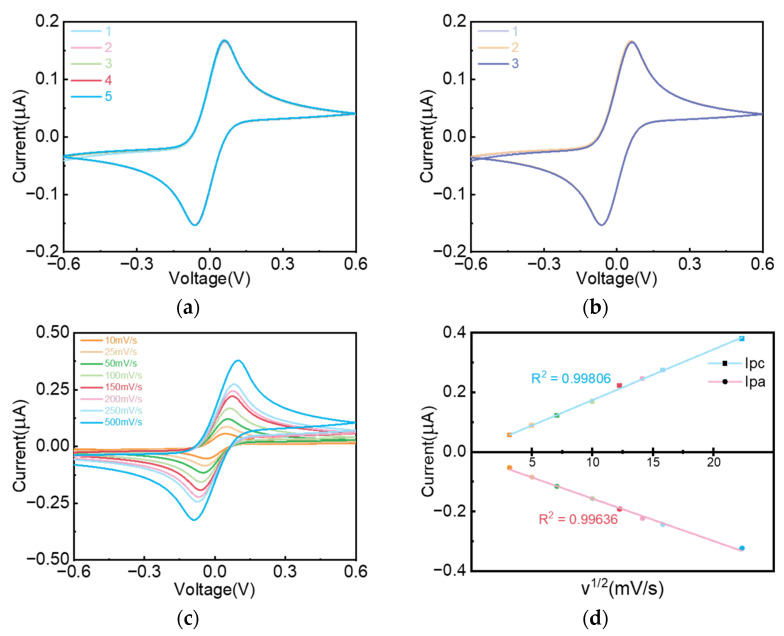
Performance characterization of electrochemical detection chip by cyclic voltammetry. (**a**) stability with five repeated measurements on the same electrode, (**b**) reproducibility with three independent electrodes, (**c**) reversibility with different scanning rates, (**d**) relationship of peak current with the square root of scan rate: cathodic curve (in blue), anodic curve (in pink).

Moreover, measurements for five cycles were carried out in 5 mM [Fe(CN)_6_]^3−/4−^ solution containing 0.1 M KCl to validate the repeatability, three cycles on different electrodes carried out to verify stability, and different scanning rates were applied to verify reversibility. The results are shown in Figure 3a–c, respectively, and indicate that the tri-channel electrodes chip had good performance in stability, repeatability, and reversibility. A disposable tri-channel electrochemical immunosensor chip was designed with the ITO-glass electrode as the bottom layer and the Ag/AgCl as the reference electrode.

### 3.2. Characterization of Immunosensor Fabrication Steps

**Figure 4:**
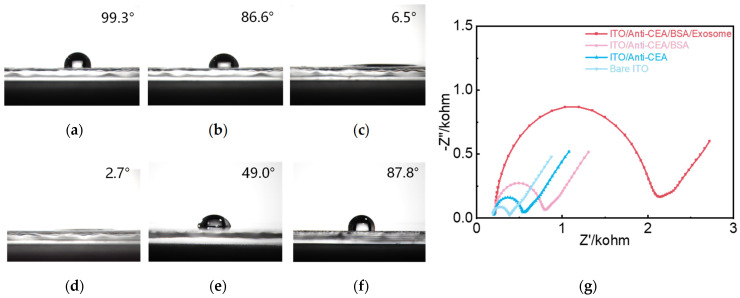
Characterization of the modification process of designed immunosensor. Water contact angle on working electrode (**a**) before cleaning, (**b**) after cleaning, (**c**) after hydroxylation, (**d**) after amination, (**e**) after aldehyde, and (**f**) after anti-CEA immobilization; (**g**) electrochemical impendence spectra of the stepwise modification of ITO/anti-CEA/BSA/exosome (frequency range 0.1 to 10 kHz).

The immobilization process included several stages: hydroxylation and silanization of disposable ITO glass electrodes, coupling of antibodies and silanized working electrode with aldehyde groups at both ends of glutaraldehyde, and blockage of the free amino terminus via BSA. Accordingly, anti-CEA was immobilized on the working electrode according to the steps shown in Scheme 1c, and the water contact angles were measured after each step of the surface modification procedures. It can be seen from Figure 4 that the surface of the working electrode exhibits a distinct hydrophilicity after hydroxylation and amination. Due to the effect of glutaraldehyde, the hydrophobicity of the surface is restored. Due to the hydrophobic effect of the antibody, the water contact angle reaches 87.8 degrees after anti-CEA immobilization. The variation of the contact angle further indicates successful stepwise modification on the working surface.

In addition, electrochemical impedance spectroscopy (EIS) was applied to further indicate the binding of ITO/anti-CEA/BSA/exosome on the working electrode, as shown in Figure 4g. The semicircle in the Nyquist plot at the high-frequency region is related to the impedance of charge transfer process in the interface of the electrode and electrolyte. An increased semicircle diameter implies the growing charge transfer resistance. The result indicated that the electrode impedance increases due to the hindrance of electron transport by protein-like insulating substances.

### 3.3. Experimental Parameters Optimization

**Figure 5:**
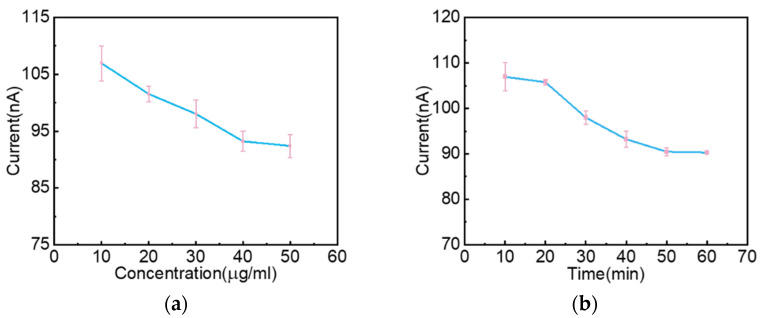
Parameter optimization. Influence of (**a**) antibody concentration used for capturing exosome on the working electrode and (**b**) incubation time of antibody on peak current responses of DPV.

The change of response current of DPV is due to the obstruction of electron transfer of the [Fe(CN)_6_]^3−/4−^, which can be used to optimize the number of antibody immobilizations related to antibody concentration and incubation time, so as to maximize the performance of the biosensor. The effect of anti-CEA concentration on immunosensor response was first examined in this study. In Figure 5a, the peak current of DPV drastically decreased with the increase of antibody concentration from 10 to 40 μg/mL, while the signal plateaued when antibody concentration continues to increase. Additionally, the effect of the anti-CEA incubation time was also studied. In Figure 5b, with the increase in incubation time, the antibody binds more tightly and showed obvious signal enhancement from 30 min to 50 min, but it tended to reach saturation after 50 min. Thus, 40 μg/mL and 50 min were chosen as the optimal values of antibody concentration and incubation time, respectively, to be used for the following detection.

### 3.4. Analytical Performance of the Designed Biosensor

#### 3.4.1. Sensitivity

**Figure 6:**
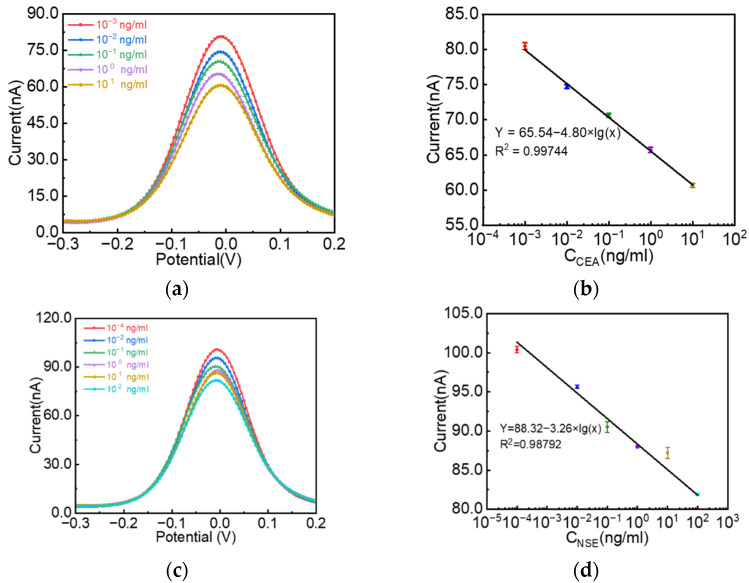

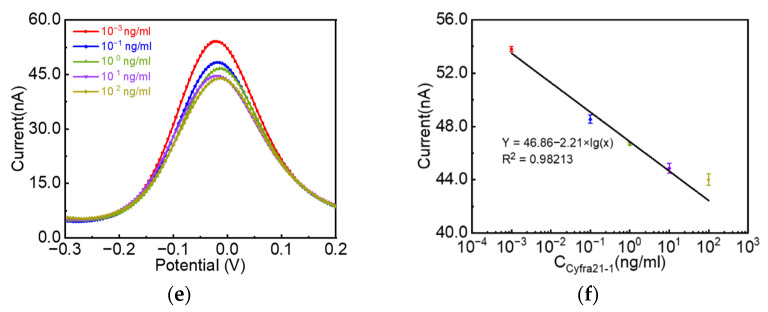
The signal responses of proposed immunosensor at different (**a**) CEA, (**c**) NSE, and (**e**) Cyfra21-1 concentrations. Linear relationship between the DPV peak current change and the logarithm of the concentrations of (**b**) CEA, (**d**) NSE, (**f**) Cyfra21-1.

A tri-channel electrochemical immunosensor chip was designed to achieve the multiple detection of the three biomarkers, CEA, NSE, and Cyfra21-1, related to lung cancer. Under optimal conditions, the immunobiosensor was prepared by immobilizing anti-CEA, anti-NSE, and anti-Cyfra21-1 on the working electrode of the chip; the detection performance of the fabricated immunosensor toward various concentrations of CEA, NSE, and Cyfra21-1 were investigated by DPV measurements. Briefly, serial gradient concentration solutions of CEA, NSE, and Cyfra21-1 were prepared with phosphate buffer solution and injected into the chip fabricated by following the protocol in Section 2.2. Antigens were detected in the concentration of 0–1 μg/mL, in which the experiment of 0 ng/mL was carried out with PBS buffer. After incubation for 30 min at 37 °C, immune complexes were formed, and then unbound biomarkers were washed off with phosphate buffer solution. Finally, DPV was performed in 5 mM K_3_[Fe(CN)_6_]/K_4_[Fe(CN)_6_]-KCl solution from −0.2 to 0.4 V. The experimental data that we have not shown indicate that the DPV response peak current was 104.4 nA when the concentration of CEA is 0 ng/mL. At a concentration of 10^−4^ ng/mL and 10^2^ ng/mL, the peak current changes were very slight, which revealed that the detection limits ad been reached. As shown in Figure 6, as the concentration of immune complexes increased, the peak value of the oxidation current of [Fe(CN)_6_]^3−^ gradually decreased, and it exhibited a logarithmic linear correlation with the concentrations. This was attributed to the immune complexes on the electrode surface as the electron communication and mass transfer barrier layer would obstruct electron transfer between [Fe(CN)_6_]^3−^ and [Fe(CN)_6_]^4−^. The standard curves of the relationship between the CEA, NSE, and Cyfra21-1 concentrations and the output current of the biosensor were obtained after linear regression. The linear detection ranges were 10^−3^ to 10 ng/mL for CEA, 10^−4^ to 10^2^ ng/mL for NSE, and 10^−3^ to 10^2^ ng/mL for Cyfra21-1, and indicated promising linear correlations, which were 0.99744, 0.98792, and 0.98213, respectively. It can be seen that the immunosensor has a wide linear range and low detection limit. Thus, high sensitivity is readily achievable within a short timescale. The excellent sensing performance of the ITO/antibody electrode suggests the suitability for exosome detection in real samples for future practical applications.

#### 3.4.2. Selectivity

**Figure 7:**
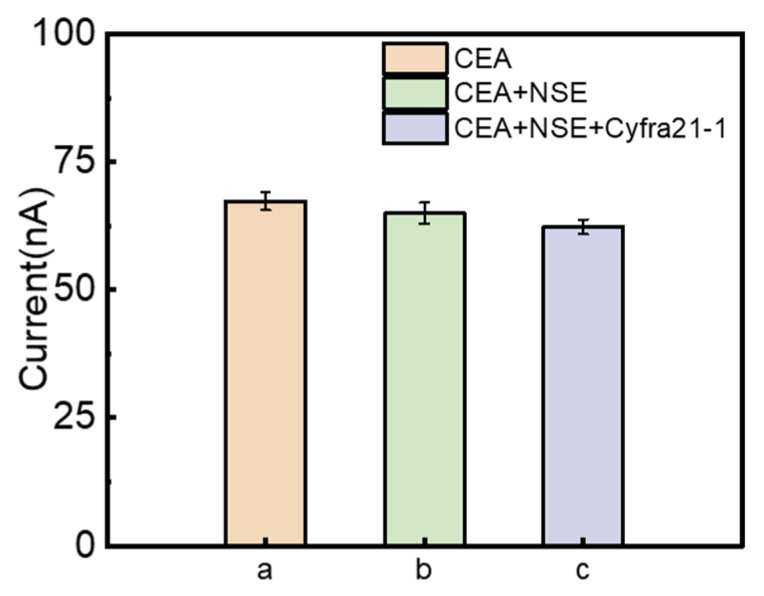
Selectivity verification in the presence of interfering protein by differential pulse voltammetry (DPV) method.

Normal samples often contain a high abundance of non-target proteins that can potentially interfere with exosome detection and lead to inaccurate results. In order to evaluate the anti-interference ability of the electrochemical immunosensor, anti-CEA was modified on the working electrode, and equal concentrations of interfering substances with NSE, NSE, and Cyfra21-1, respectively, were added to the CEA solution (1 ng/mL). The DPV response peak current was 67.35, 64.93, and 64.02 nA, respectively. As can be seen from Figure 7, the peak current was decreased due to the addition of interfering substances, which was due to the obstruction of electron transmission caused by nonspecific binding. In general, the differences of the peak values of the response current are less than 5%, which is within the allowable error range [33].

#### 3.4.3. Storage Stability

**Figure 8:**
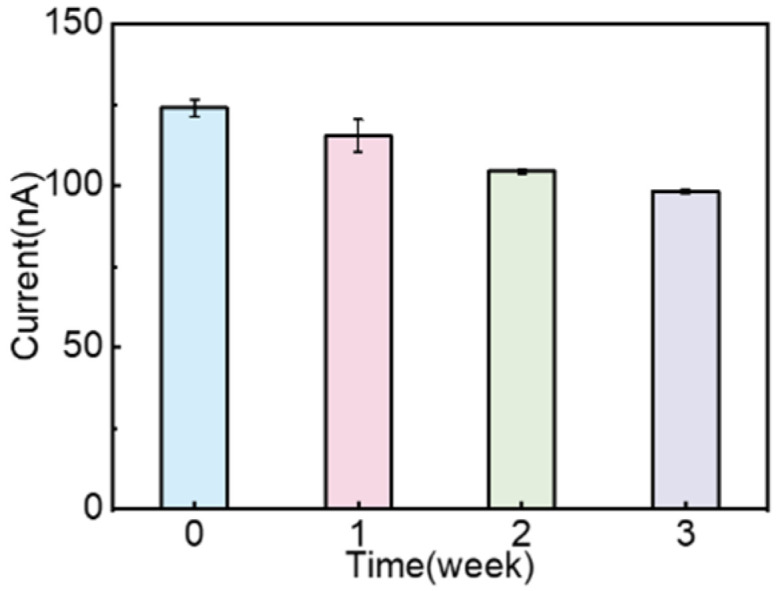
Stability verification stored in PBS at 4 °C in three weeks by differential pulse voltammetry (DPV) method.

The storage stability of the detection method is very important in practical application. In order to evaluate the storage stability of the electrochemical sensor chip, the pre-constructed anti-CEA immunosensor was stored at 4 °C in 10 mM PBS (pH = 7.4) for 1, 2, and 3 weeks, and the electrochemical response signal value was recorded. As shown in Figure 8, an approximately 19% reduction in current change was obtained, which was due to the slight degradation of the protein after long-term storage at 4 °C, which was still within the acceptable range. The prepared immunosensor should be used as soon as possible to avoid interference.

As noted above, the present sensor has excellent performance in sensitivity, anti-interference ability, and storage stability. 

### 3.5. Characterization of Exosome

**Figure 9:**
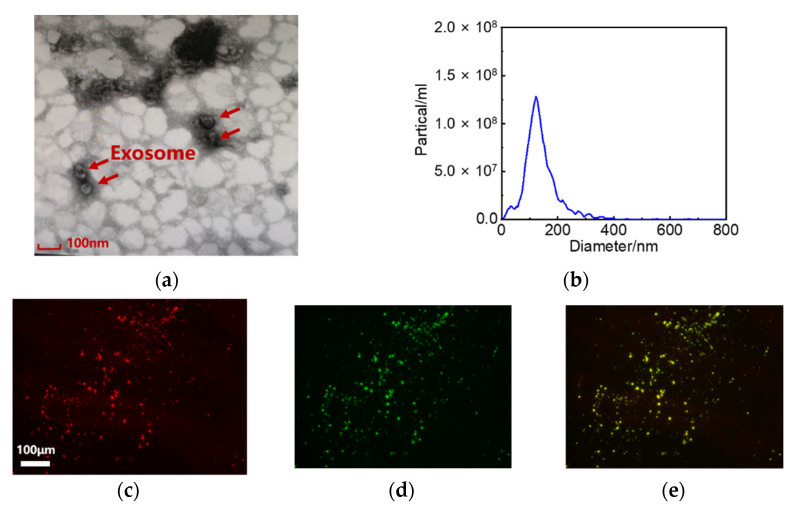
The characterization of exosome. (**a**) TEM image and (**b**) ZetaView analysis of the size distribution of extracted exosomes derived by H1395 cells; (**c**) Under 549 nm laser excitation, red fluorescence represents antibodies, (**d**) Under 488 nm laser excitation, green fluorescence represents exosomes, (**e**) Merged picture of (**c**,**d**).

We extracted exosomes from lung cancer cells according to the protocol in Section 2.4.2. Morphological characterization of exosomes fixed on the copper grid by TEM are demonstrated in Figure 9a. It can be seen that the exosomes showed a saucer-like morphology with clear outline. The mean particle size distribution and concentration of exosomes was further characterized by NTA. Figure 9b revealed that the mean size of exosomes was 120 ± 80 nm within expectation, and the concentration was 1.5 × 10^7^ particles/mL. Moreover, to visualize the immobilization of antibody on the surface of ITO and the immune binding of exosomes and cy3-anti-NSE, exosomes was pre-labeled with a fluorescent lipid probe (Dio) that will specifically stain the cell membrane only. The Cy3 dye is a red fluorophore upon excitation and can be captured by fluorescent camera, while the labeled exosomes showed green fluorescence dots under blue light excitation. As can be seen from Figure 9c,d the antibody is modified to the surface of the working electrode and can be used to capture exosomes. The result of the combination is shown in Figure 9e, which presents an excellent bonding effect.

### 3.6. Multiplexed Detection of Exosome Biomarkers of Lung Cancer

**Figure 10:**
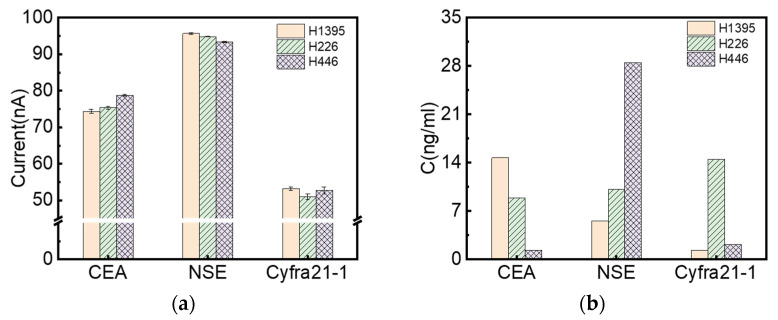
Quantitative detection of markers of exosomes extracted from H1395, H226, and H446 lung cancer cells, respectively. DPV response (**a**) and concentration expression (**b**) of three markers of exosomes measured in different cell lines.

Pathologically, lung cancer is classified into small-cell lung carcinoma (SCLC) and non-small-cell lung carcinoma, which comprise around 85% and 15% of all cases, respectively. The latter can be further divided into lung adenocarcinoma, lung squamous cell carcinoma, and others [40]. Exosome tumor markers hold promise for the early detection and diagnosis of lung cancer in the current applications [41]. However, the detection of a single tumor marker is limited to sensitivity and accuracy, and the classical protein markers of exosomes are limited to specificity. Accordingly, we analyzed the role of three exosome biomarkers, CEA, NSE, and Cyfra21-1, in lung cancer diagnosis. Briefly, a tri-channel electrochemical detection chip was prepared by immobilizing anti-CEA, anti-NSE, and anti-Cyfra21-1 successively on the working electrodes. Exosome samples extracted from H1395 (lung adenocarcinoma), H226 (lung squamous cell carcinoma), and H466 (small-cell lung carcinoma) lung cancer cell lines were diluted (1000-fold) with PBS and then injected into the tri-channel electrochemical chip. After incubation at 37 °C for 30 min, the chip was washed with buffer and subsequently detected by DPV in 5 mM [Fe(CN)_6_]^3−/4−^ solution containing 0.1 M KCl. As the protein concentration of exosomes was increased, peak current gradually decreased. The peak value of the sample was converted to analyze the concentration using the standard curve mentioned above. The expression of CEA, NSE, and CYFRA21-1 in three kinds of lung cancer cells is shown in Figure 10. In H1395, H226, and H446 lung cancer cells, the contents of exo-CEA were 14.7 ng/mL, 8.9 ng/mL, and 1.35 ng/mL; the contents of exo-NSE were 5.58 ng/mL, 10.19 ng/mL, and 28.51 ng/mL; the contents of exo-Cyfra21-1 were 1.32 ng/mL, 14.5 ng/mL, and 2.16 ng/mL, respectively. Different lung cancer cells showed significantly different expression levels of exosome biomarkers. For example, exo-CEA was abundant in lung adenocarcinoma and exo-NSE was significantly increased in small cell lung cancer, while exo-Cyfra21-1 was prominent in lung squamous cell carcinoma, which may provide the possibility for further tumor classification.

## 4. Conclusions

In summary, a tri-channel electrochemical immune detection system was developed for enzyme-free and label-free detection of multiple exosome biomarkers (CEA, NSE, and Cyfra21-1) for early diagnosis of lung cancer. The fabricated biosensor performed a linear response with a wide range and a good selectivity and stability. Highlights summarized from this work are as follows:

1. The utilization of ITO glass instead of precious metals such as gold offers an inexpensive, simple, and sensitive system, which makes it easy to realize small sample amounts and integrated detection when combined with microchannels.
2. The proposed immunosensor with optimized parameters can detect exosome markers in a range from 10^−3^ to 10 ng/mL for CEA, 10^−4^ to 10^2^ ng/mL for NSE, and 10^−3^ to 10^2^ ng/mL for Cyfra21-1, with a detection limit below 10^−4^ ng/mL, which was lower than the conventional ELISA method. The immunosensor is suitable for exosome detection in real samples for practical applications.
3. The combined detection of multiple exosome markers has a higher efficiency than a single biomarker of ELISA. The difference in expression level may guide the typing of lung cancer; nevertheless, clinical trials with adequate sample volume are still needed for further validation.

## Data Availability

The data presented in this study are available upon request from the corresponding author.
